# Low levels of IgM antibodies recognizing oxidation-specific epitopes are associated with human non-alcoholic fatty liver disease

**DOI:** 10.1186/s12916-016-0652-0

**Published:** 2016-07-22

**Authors:** Tim Hendrikx, Martin L. Watzenböck, Sofie M. A. Walenbergh, Shahzada Amir, Sabrina Gruber, Maria Ozsvar Kozma, Heike I. Grabsch, Ger H. Koek, Marieke J. Pierik, Katharina Staufer, Michael Trauner, Satish C. Kalhan, Daisy Jonkers, Marten H. Hofker, Christoph J. Binder, Ronit Shiri-Sverdlov

**Affiliations:** Departments of Molecular Genetics, Pathology, and Internal Medicine, Division of Gastroenterology and Hepatology, School of Nutrition and Translational Research in Metabolism (NUTRIM) and School of Oncology and Developmental Biology (GROW), Maastricht University and Maastricht University Medical Center (MUMC), PO Box 616, 6200 MD Maastricht, The Netherlands; Departments of Laboratory Medicine, Surgery, and Internal Medicine III, Division of Gastroenterology And Hepatology, Medical University of Vienna, Vienna, Austria; Center for Molecular Medicine (CeMM) of the Austrian Academy of Sciences, Vienna, Austria; Department of Pathobiology, Lerner Research Institute, The Cleveland Clinic Foundation, Cleveland, OH USA; Department of Pathology & Laboratory Medicine, University Medical Center Groningen, University of Groningen, Groningen, The Netherlands

**Keywords:** Non-alcoholic steatohepatitis, Lipid oxidation, Adaptive immune response, Fatty liver, IgM

## Abstract

**Background:**

Lipid oxidation of membrane phospholipids is accompanied by the formation of oxidation-specific epitopes (OSE). These epitopes are recognized by specific antibodies and represent danger-associated molecular patterns that are generated during chronic inflammatory processes. In a murine model for hepatic inflammation during non-alcoholic fatty liver disease (NAFLD), increased antibody levels targeting OSE were found to be protective. Here, our aim was to determine an association between OSE-specific antibody titers and NAFLD in humans.

**Methods:**

IgM and IgG levels with specificity for various OSE were assessed in the plasma of patients with NAFLD (*n =* 71) and healthy controls (*n =* 68). Antibody titers were comprehensively analyzed in patients with NAFLD after classification by histological evaluation of liver biopsies. Statistical analysis was performed to determine significant correlations and odds ratios. To study the specificity for NAFLD, plasma antibody titers were measured in patients with hepatitis C (*n* = 40) and inflammatory bowel disease (*n =* 62).

**Results:**

IgM titers against OSE were lower in patients with NAFLD compared to controls. Further biopsy-based classification of patients with NAFLD did not show any difference in IgM levels. Plasma IgM titers towards the P1 mimotope demonstrated an inverse correlation with markers for obesity, systemic inflammation, and liver damage. In contrast, hepatitis C and increased disease activity during inflammatory bowel disease was not associated with reduced IgM titers.

**Conclusions:**

Our data highlight the importance of immune recognition of OSE by IgM antibodies in the pathophysiology of NAFLD.

**Electronic supplementary material:**

The online version of this article (doi:10.1186/s12916-016-0652-0) contains supplementary material, which is available to authorized users.

## Background

Non-alcoholic fatty liver disease (NAFLD) constitutes a spectrum of liver diseases characterized by hepatic lipid accumulation (steatosis), which when combined with hepatic inflammation is known as non-alcoholic steatohepatitis (NASH). Steatosis is generally considered to be a benign and reversible condition that is present in about two thirds of patients with the metabolic syndrome [1]. The presence of inflammation in a fatty liver is thought to drive disease progression, including the development of fibrosis, cirrhosis, or hepatocellular carcinoma, ultimately requiring liver transplantation [2]. The mechanisms leading to hepatic inflammation are currently not fully understood. Hence, therapy options are limited and accurate, early, non-invasive diagnostic tools are lacking [3].

Immune reactions triggered by oxidative stress have been shown to be involved during the progression of NAFLD to fibrosis [4]. Moreover, oxidized low-density lipoproteins (OxLDL) are known to play a major role in atherosclerosis and various other related metabolic disturbances, such as NASH [5]. The oxidation of LDL is accompanied by the formation of various epitopes on the surface of OxLDL, called oxidation-specific epitopes (OSE) [6]. These epitopes include protein adducts with lipid peroxidation breakdown products, such as malondialdehyde (MDA), malondialdehyde-acetaldehyde (MAA), and 4-hydroxynonenal, as well as with phosphocholine (PC)-containing oxidized phospholipids [6]. OSE on the surface of OxLDL are recognized by both innate and acquired humoral immunity, including specific antibodies that are found in the plasma of humans and animal models [6, 7]. Evidence for a protective role of OSE-specific immunoglobulin M (IgM) antibodies in NASH is based on our findings that increased IgM levels protect from hepatic inflammation in hyperlipidemic mice [8]. To summarize, immunized *Ldlr*^*−/−*^ mice with heat-inactivated pneumococci, which induce the production of anti-OxLDL IgM due to molecular mimicry with OxLDL, had less hepatic inflammation than control mice after a Western diet. Moreover, we recently showed that increased levels of IgM antibodies as a result of Siglec-G deficiency inhibits diet-induced hepatic inflammation and atherosclerosis in *Ldlr*^*−/−*^ mice [9]. However, the relationship between plasma antibody levels targeting OSE and hepatic inflammation during NAFLD has not been investigated in humans so far.

Here, we aimed to determine an association between plasma antibodies targeting OSE and NAFLD in humans. For this purpose, IgM and IgG antibody titers against different model epitopes of oxidized lipids were measured in the plasma of patients with NAFLD and compared to those in control subjects. Liver biopsies from patients with biopsy-proven NASH were examined for the presence of MDA epitopes by immunohistochemistry. Additionally, to test the specificity of our findings for NAFLD, antibody levels were determined in two cohorts consisting of patients with hepatitis C and inflammatory bowel disease (IBD). Our data highlight the importance of immune recognition of OSE by IgM antibodies in NAFLD and suggest that low IgM levels against end products of lipid oxidation during fatty liver disease are a consequence of obesity.

## Methods

### Antigens and antibodies

Human MDA-LDL, MAA-LDL, CuOx-LDL, and PC-BSA were prepared as previously described [10]. MDA2 (a kind gift from Dr J. L. Witztum, San Diego, CA, USA) is a murine IgG monoclonal antibody against MDA-lysine epitopes. Linear peptide P1 (HSWTNSWMATFL) was purchased from Peptide 2.0 Inc. (Chantilly, VA, USA).

### Immunohistochemistry

Immunohistochemistry for MDA using the monoclonal antibody MDA2 was performed on 10 liver biopsies diagnosed as NASH, originating from the Maastricht Pathology Tissue Collection. Collection, storage, and use of tissue and patient data were performed in agreement with the “Code for Proper Secondary Use of Human Tissue in the Netherlands” (http://www.federa.org). The staining was performed as described previously (antibody dilution 1:2000) and biopsies were evaluated for the presence of MDA [10].

### Chemiluminescent ELISA

Chemiluminescent ELISA was performed as previously described [10]. In brief, purified anti-human IgM (BD Pharmingen, San Jose, CA, USA) and antigens (MDA-LDL, MAA-LDL, CuOx-LDL, PC-BSA) at concentrations of 5 μg/ml in 50 μl phosphate-buffered saline (PBS)-EDTA were added to each well of a 96-well white, round-bottom microtitration plate (MicrofluorII roundbottom; Thermo, Rochester, New York, USA) and incubated overnight at 4 °C. P1 peptide (Peptide 2.0 Inc.) was coated at 10 μg/ml in NaHCO_3_ (pH 8.5) coating buffer and incubated overnight at 4 °C. After washing and blocking with Tris-buffered saline (TBS) with or without EDTA (pH 7.4, containing 1 % bovine serum albumin (BSA), 30 min at room temperature), the plate was incubated with plasma samples in their respective dilutions (total IgM: 1:40,000; MDA-LDL, MAA-LDL, P-1, Cu-OxLDL, PC-BSA IgM/IgG: 1:200) in 1 % BSA in TBS with EDTA (pH 7.4) for 2 h at room temperature or overnight at 4 °C. Alkaline phosphatase (AP)-labeled goat anti-human IgM (μ-chain specific; Sigma-Aldrich, Vienna, Austria; 1:50,000 in TBS-BSA) or AP-labeled goat anti-human IgG (γ-chain specific; Sigma-Aldrich, Vienna, Austria; 1:50,000 in TBS-BSA) was used for detection. AP-conjugated secondary reagents were detected using Lumi-Phos (Lumigen, Southfield, Michigan, USA; 33 % solution in water) and a Synergy 2 Luminometer (BioTek, Winooski, Vermont, USA). Washing steps were performed on an ELx405 Select Deep Well Microplate Washer (BioTek, Winooski, Vermont, USA) with PBS or PBS-EDTA. Internal controls were included on each microtiter plate to detect potential variations between microtiter plates. The intra-assay coefficients of variation for all assays were 5–15 %.

### NAFLD cohort for antibody measurements

Healthy controls (*n* = 68) were recruited by advertisement and patients classified as NAFLD (*n* = 53) were recruited from the metabolic clinics of the Cleveland Clinic and MetroHealth Medical Center in Cleveland, OH, USA. The study protocol was approved by the Institutional Review Boards at the Cleveland Clinic and MetroHealth Medical Center. A detailed clinical history was obtained for each participant, followed by a physical examination. Hepatic ultrasonography was performed on all participants by the same investigator (S. Dasarathy) in order to confirm the absence of steatosis in controls. Moreover, plasma transaminase levels were determined in all participants. Where steatosis and increased plasma transaminase levels were detected, a liver biopsy was taken for further characterization of NAFLD. Liver biopsies from patients were reviewed in a blinded manner by the same pathologist and NASH was defined as an NAFLD activity score ≥4 and a histologic diagnosis of NASH by the pathologist. Participants with other forms of chronic liver disease were excluded by screening for autoimmune liver disease (autoimmune hepatitis, primary biliary cirrhosis, sclerosing cholangitis), for metabolic liver disease (hemochromatosis, Wilson’s disease), for alpha-1 antitrypsin deficiency, and for viral hepatitis (hepatitis B and hepatitis C). Participants with a history of daily alcohol intake (women >20 g, men >30 g), intravenous drug use, or a history of bowel surgery were also excluded. None of the participants were on any medications known to cause hepatic steatosis or taking vitamin supplements. Written informed consent was obtained from all participants after having the procedure fully explained. Blood and liver sampling was done as described before [11–13]. Plasma samples were used to perform the antibody determinations. Relevant clinical data, including gender, age, weight, and height, together with inflammatory and liver blood test results, were collected at the time of liver biopsy and are represented in Table 1. The body mass index (BMI) was calculated using the formula: weight (kg)/[height (m)]^2^. Further clinical and biochemical data for all patients are summarized in Table 1 (Additional file 1).

**Table 1:** Clinical and biological characteristics of patients with non-alcoholic steatohepatitis for antibody measurements

	Participant characteristics (mean ± SD)		
	Controls (*n =* 68)	NAFLD (simple steatosis) (*n =* 18)	NAFLD (NASH) (*n =* 53)
Male/female	27/41	9/9	24/29
Age (y)	40.9 ± 12	45.7 ± 13	45.9 ± 10
BMI (kg/m^2^)	26.3 ± 4.9	33.6 ± 5.6	34.3 ± 4.7
Waist circumference (cm)	89.4 ± 13.7	108.5 ± 12.9	109.0 ± 14.3
Triglycerides (mg/dL)			
Median	75	151	160
Interquartile range	48	66	118.5
Cholesterol (mg/dL)	181.33 ± 32.9	197.8 ± 53.2	196.2 ± 43.5
HDL (mg/dL)	54.8 ± 17.7	41.44 ± 8.0	42.87 ± 8.9
LDL (mg/dL)	105.65 ± 26.9	135.3 ± 47.2	122.9 ± 36.3
	Markers of inflammation and liver blood tests (mean (IQR))		
TNF-α (pg/ml)	2.5 (0.7)	3 (2.3)	3 (1.9)
MCP-1 (pg/ml)	96.9 (27)	122.4 (24.05)	120.1 (37.95)
IL-1β (pg/ml)	0.42 (0.87)	0.27 (0.36)	0.17 (0.64)
IL-6 (pg/ml)	13.2 (35.8)	16.1 (8.1)	24.5 (45.25)
IL-8 (pg/ml)	5.4 (4)	5.34 (3.5)	7.7 (8.05)
γGT (IU/L)	-	51.6 (22.7)	55.6 (32)
Leptin	11.1 (8.8)	23.0 (15.8)	30.5 (18.5)
AST (IU/L)	21 (9)	28 (11.8)	49 (56.5)
ALT (IU/L)	18 (8)	38 (22.8)	66 (99.5)
AST/ALT ratio	1.3 (0.25)	0.8 (0.2)	0.8 (0.4)

*AST* aspartate transaminase, *ALT* alanine transaminase, *BMI* body mass index, *γGT* gamma-glutamyltransferase, *HDL* high-density lipoprotein, *IL* interleukin, *IQR* interquartile range, *LDL* low-density lipoprotein, *MCP-1* monocyte chemoattractant protein-1, *NAFLD* non-alcoholic fatty liver disease, *NASH* non-alcoholic steatohepatitis, *TNF-*α tumor necrosis factor alpha

### Hepatitis C cohort

Healthy controls (*n* = 20) were recruited by advertisement and patients with chronic hepatitis C (*n* = 20) were recruited from the Department of Internal Medicine III, Division of Gastroenterology and Hepatology, Medical University of Vienna, Austria. The study protocol was approved by the Institutional Review Boards at the Medical University of Vienna. Hepatic ultrasonography and liver biopsy was performed on all participants prior to scheduled antiviral therapy. Moreover, plasma transaminase levels were determined in all participants. Liver biopsies of patients were reviewed in a blinded manner by two independent pathologists. Participants with other forms of chronic liver disease or with a history of daily alcohol intake were excluded by screening. Written informed consent was obtained from all participants after having the procedure fully explained. Further clinical data for the patients are summarized in Table 2 (Additional file 2).

**Table 2:** Clinical characteristics of patients with hepatitis C

	Participant characteristics (mean ± SD)	
	Controls (*n =* 20)	Hepatitis C (*n =* 20)
Male/female	12/8	11/9
Age (y)	48.4 ± 14	48.2 ± 12
Triglycerides (mg/dL)	95.1 ± 52.3	111.25 ± 57.3
Cholesterol (mg/dL)	197.05 ± 31	162.6 ± 40.9
HDL (mg/dL)	71.2 ± 14.5	43.2 ± 16.2
LDL (mg/dL)	106.83 ± 27.1	97.1 ± 38.5
γGT (IU/L)	18.5 ± 6.5	265.1 ± 344.1
AST (IU/L)	24.9 ± 13	113.7 ± 94
ALT (IU/L)	22.7 ± 10.4	127.1 ± 86.3
AST/ALT ratio	1.13 ± 0.27	0.95 ± 0.55

*AST* aspartate transaminase, *ALT* alanine transaminase, *γGT* gamma-glutamyltransferase, *HDL* high-density lipoprotein, *LDL* low-density lipoprotein

### Patients with inflammatory bowel disease

Between September 2009 and December 2010, consecutive patients with IBD were recruited during routine follow-up at the Gastroenterology Outpatient Clinic of the MUMC+ and participated in a prospective 1-year follow-up study [14]. All patients had an established diagnosis of IBD based on clinical, endoscopic, histological, and/or radiological criteria. Demographic and clinical data were obtained using the MUMC+ computer-based medical registration database. Disease phenotype at the time of diagnosis was determined by the Montreal classification [15]. The study was approved by the Medical Ethics Committee of MUMC+ and written informed consent was obtained prior to participation. A subset of patients who underwent an endoscopy for clinical reasons was included for the present study. Disease activity was assessed during endoscopy by an experienced gastroenterologist (MP) as inactive (0), mild (1), moderate (2), or severe (3) (Additional file 3).

### Statistical analysis

Continuous variables are presented as means and standard deviations or as medians and interquartile ranges for skewed distributions. Categorical data are presented as absolute frequencies and percentages. Student’s *t*-test or the Mann–Whitney *U* test (for non-normally distributed data) was used to compare a variable between two subgroups. Multivariate logistic regression models were used to assess the association between P1-specific IgM levels and NAFLD in the specific cohort after adjusting for age, gender, BMI, and total IgM levels. All reported *p*-values are two-tailed and considered significant at *p <* 0.05. Statistical procedures were carried out using Graphad Prism 6 or IBM SPSS 20.

## Results

### MDA epitopes are present in livers of patients with NASH

**Fig. 1:**
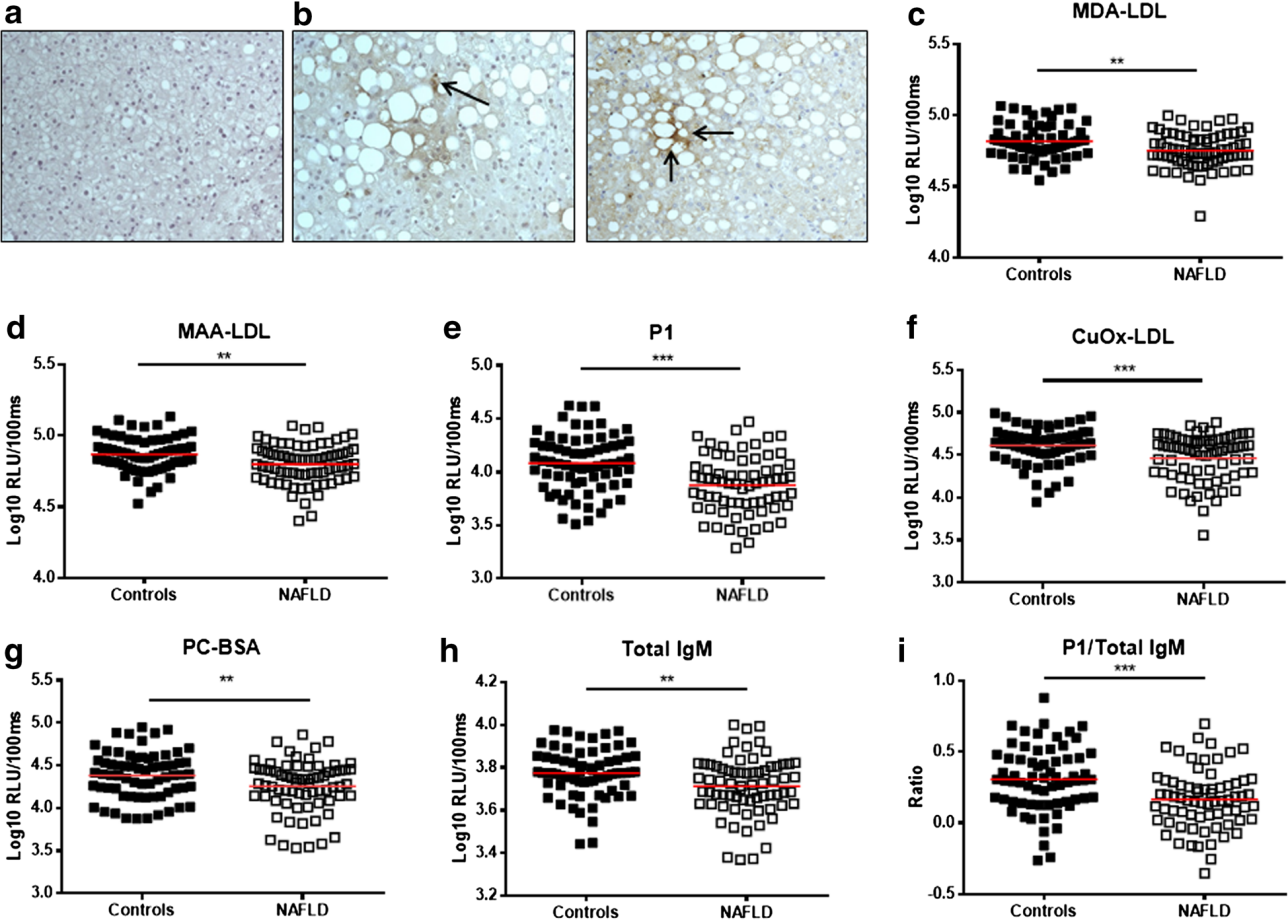
Malondialdehyde (*MDA*) epitopes in livers of patients with non-alcoholic steatohepatitis (*NASH*) and relationship between plasma immunoglobulin M (*IgM*) titers and non-alcoholic fatty liver disease (*NAFLD*). Immunohistochemical detection of MDA epitopes in liver sections (magnification 20×) of **a** a negative control and **b** two patients with NASH – arrows indicate localization of MDA2-positive staining in the liver tissue. Plasma IgM antibody titers against **c** MDA-low-density lipoprotein (*LDL*), **d** malondialdehyde-acetaldehyde (*MAA*)-LDL, **e** P1 mimotope, **f** CuOx-LDL, **g** PC-BSA, and **h** total IgM levels in patients with NAFLD and healthy controls. **i** Ratio of anti-P1 IgM titers to total IgM levels in patients with NAFLD and controls. Data are expressed in log10 relative light units (*RLU*)/100 ms. *p < 0.05, **p < 0.01, ***p < 0.001

To determine whether OSE are generated during the spectrum of NAFLD, immunohistochemical staining for MDA epitopes with the monoclonal antibody MDA2 was performed on liver biopsies of patients with biopsy-proven NASH. As illustrated in Fig. 1a–c, small clusters of MDA2-positive staining were detected between swollen hepatocytes of liver biopsies from patients with NASH, indicating the presence of MDA epitopes.

### IgM antibody titers towards different OSE are lower in patients with NAFLD

To investigate the potential relationship between antibodies specifically targeting epitopes of OxLDL and NAFLD, IgM and IgG antibody titers against MDA, MAA (an immunodominant advanced MDA-lysine adduct), P1 (a highly specific peptide mimotope of MAA [10]), Cu-OxLDL, and PC-BSA were measured in the plasma of patients with NAFLD (*n =* 71) and control participants with confirmed absence of steatosis (*n =* 68). The values of the different measurements were tested for normality with Shapiro–Wilks test and the results indicated that data were not normally distributed for most of the measurements in either of the groups (Additional file 4: Table S1). Therefore, all data were logarithmically transformed (log10) and represented using a log10 scale in Fig. 1. The observed relative lights units can be found in Additional file 4: Table S2.

In line with our expectations, patients with NAFLD had significantly lower IgM titers than controls against MDA-LDL (4.755 versus 4.819 log10 RLU/100 ms, *p =* 0.0019; Fig. 1c, Additional file 4: Table S2), MAA-LDL (4.801 versus 4.868 log10 RLU/100 ms, *p =* 0.0026; Fig. 1d, Additional file 4: Table S2), P1 (3.877 versus 4.08 log10 RLU/100 ms, *p <* 0.0001; Fig. 1e, Additional file 4: Table S2), CuOx-LDL (4.46 versus 4.612 log10 RLU/100 ms, *p =* 0.0004; Fig. 1f, Additional file 4: Table S2), and PC-BSA (4.255 versus 4.383 log10 RLU/100 ms, *p =* 0.0096; Fig. 1g, Additional file 4: Table S2). No differences were found in specific IgG antibody titers towards the different epitopes of OxLDL in patients with NAFLD compared to controls (Additional file 4: Table S3).

Interestingly, total IgM antibodies were also significantly lower in patients with NAFLD compared to healthy participants (3.773 versus 3.712 log10 RLU/100 ms, *p =* 0.0044; Fig. 1h, Additional file 4: Table S2). Notably, IgM titers towards the specific P1 mimotope, which showed the strongest reduction in patients with NAFLD compared to controls, remained significant after adjusting for total IgM levels (0.3074 versus 0.1658 (ratio), *p =* 0.0002, Fig. 1i). These data point towards a negative association between IgM antibodies and oxidized lipids and fatty liver disease, and indicate an important role for lipid oxidation in NAFLD.

### Plasma anti-P1 IgM levels are reduced in the early phase of NAFLD and are not influenced by inflammation or fibrosis

**Fig. 2:**
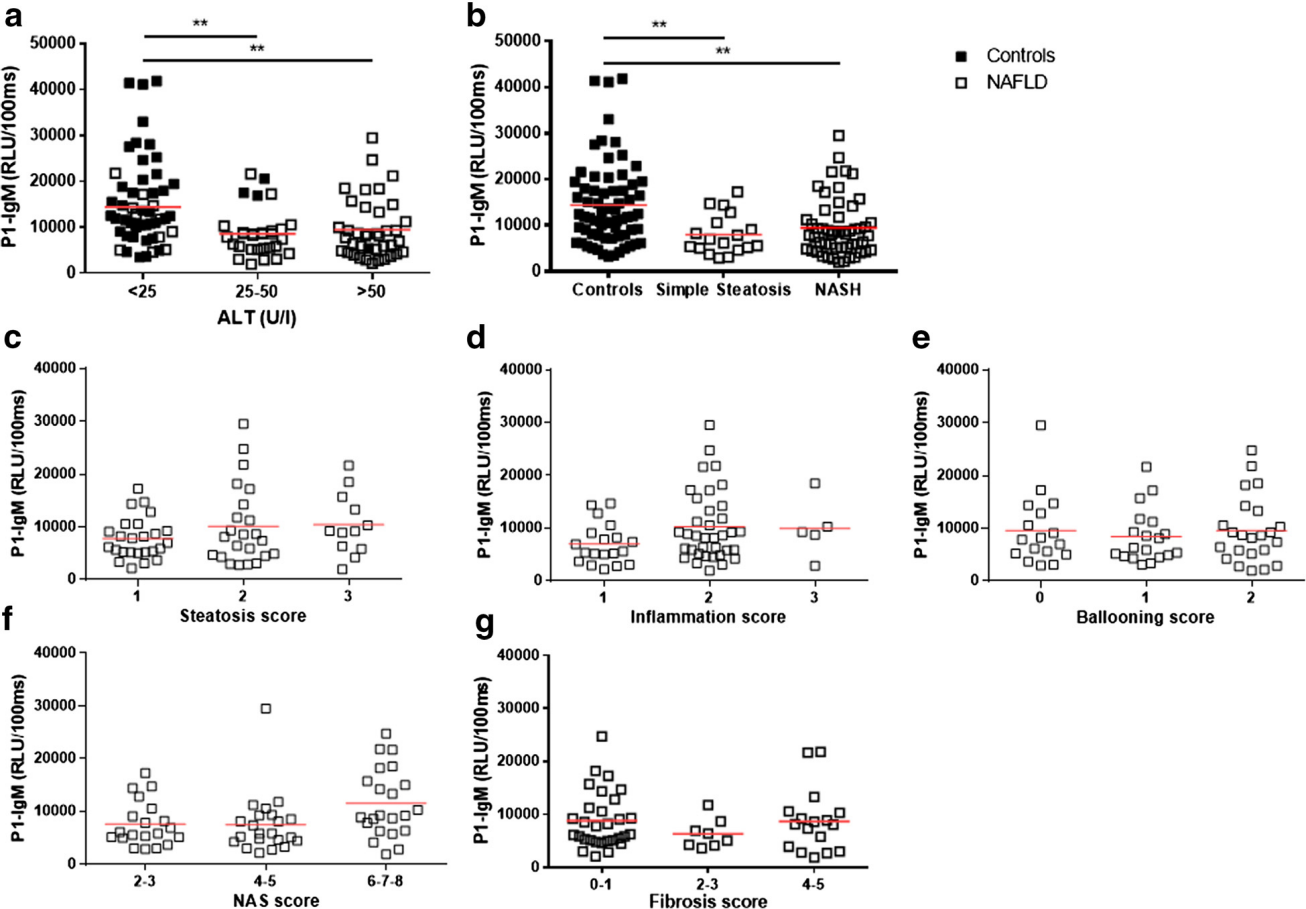
Relationship between plasma P1-immunoglobulin (*IgM*) levels and non-alcoholic fatty liver disease (*NAFLD*) upon further characterization of liver biopsies. **a** IgM titers towards P1 in controls and patients with NAFLD grouped based on their ALT levels. **b** Plasma IgM antibody titers against the P1 mimotope in patients with NAFLD in which the liver biopsy was evaluated and scored as simple steatosis versus non-alcoholic steatohepatitis (*NASH*). Anti-P1 IgM titers after scoring liver biopsies for **c** steatosis, **d** inflammation, **e** hepatocyte ballooning, **f** applying the NAFLD activity score (*NAS*), and **g** scoring fibrosis. Data are expressed in relative light units (*RLU*)/100 ms and presented as means. **p < 0.01

Because anti-P1 IgM levels showed the strongest reduction in patients with NAFLD and due to its high clinical reproducibility, further analyses were conducted using the anti-P1 IgM measurements. To determine in which phase in the disease spectrum of NAFLD that plasma IgM titers towards P1 are reduced, participants were divided according to their ALT levels (<25, 25–50, >50 IU/L) because a normal ALT level is expected to be <50 IU/L. Interestingly, P1-IgM levels were reduced in participants with moderately elevated ALT levels (25–50 IU/L) or high ALT levels (>50 IU/L) when compared with the P1-IgM levels of participants with ALT levels <25 IU/L (Fig. 2a). Moreover, more than 90 % of control participants were classified as having low ALT levels. These data suggest that IgM titers are already lower in early phases of NAFLD, before liver damage is detected based on ALT levels.

Next, we evaluated the relationship between IgM to P1 and different classifications based on histological scoring of liver biopsies taken from patients with NAFLD. First, the potential of anti-P1 IgM to distinguish simple steatosis from NASH was tested. Whereas IgM titers were lower in NAFLD compared with controls, no difference was observed in specific IgM levels between patients with steatosis and NASH (Fig. 2b). Moreover, no differences were observed in plasma P1-IgM titers after classifying the patients according to a score for steatosis (Fig. 2c), inflammation (Fig. 2d), hepatocyte ballooning (Fig. 2e), the NAFLD activity score (NAS) (Fig. 2f), or for fibrosis (Fig. 2g). These data further suggest that antibodies recognizing oxidized lipids are involved during early processes in the development of NAFLD.

### Low IgM titers against the P1 mimotope have a high predictive value for the presence of NAFLD

**Fig. 3:**
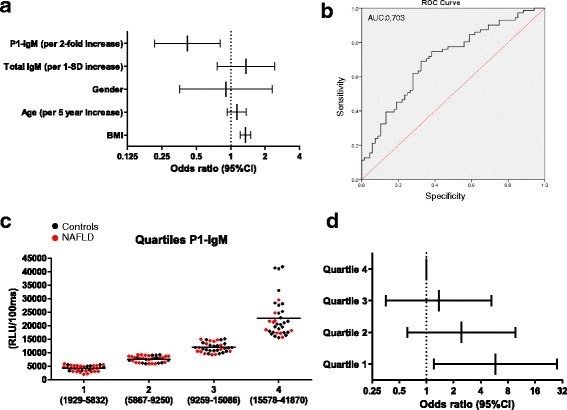
Single logistic regression model, odds ratio determination, and receiver operating characteristic (*ROC*) curve analysis for P1-specific IgM levels in non-alcoholic fatty liver disease (*NAFLD*). **a** Odds ratios for the predictive power of anti-P1 immunoglobulin (*IgM*) titers (log2 scale) for fatty liver disease after adjustment for age, gender, body mass index (*BMI*), and total IgM. **b** ROC curve analysis for the diagnosis of NAFLD with P1-IgM. **c** IgM titers towards P1 were divided into quartiles (*black*: controls; *red*: NAFLD) and **d** the odds ratios P1-IgM (log2 scale) for NAFLD were computed across these quartiles after adjusting for age, gender, BMI, and total IgM levels. *CI* confidence interval

To determine the power of low plasma IgM titers against the P1 mimotope to predict the presence of fatty liver disease, age, gender, BMI, total IgM levels, and anti-P1 IgM levels were combined in a single logistic regression model. To account for skewness in their distribution, base-2 logarithms of P1-specific IgM levels were used in the model. Thus, adjusted odds ratios (OR) for this variable reflect the change in odds for an increase of one log2 (the equivalent of a doubling of the value) in the measure. Without adjustment for other risk factors, age (OR = 1.206; confidence interval = 1.039–1.4), BMI (OR = 1.353; confidence interval = 1.228–1.491), total IgM (OR = 0.619; confidence interval = 0.431–0.888), and P1-IgM (OR = 0.428; confidence interval = 0.282–0.65) were all significant predictors for the presence of NAFLD, and female gender was not (Additional file 4: Table S4). Importantly, P1-IgM levels (OR = 0.419; confidence interval = 0.216–0.813) were still a significant predictor for the presence of NAFLD after adjustment for age, gender, BMI, and total IgM (Fig. 3a, Additional file 4: Table S5). Moreover, adjusting for waist circumference instead of BMI in our model did not change the significance for anti-P1 IgM levels (OR = 0.432; confidence interval = 0.261–0.813) in predicting the presence of NAFLD (Additional file 4: Table S6 and Additional file 5: Figure S1). In line with our expectations, BMI (OR = 1.343; confidence interval = 1.211–1.49) and waist circumference (OR = 1.666; confidence interval = 1.354–2.049) were still significant predictors for NAFLD after adjustment, whereas other variables lost their significance (Additional file 4: Tables S5 and S6).

A receiver operating characteristic (ROC) curve analysis was performed to assess the diagnostic value of anti-P1 IgM titers for NAFLD. An area under the curve (AUC) of 0.703 (confidence interval: 0.617–0.790) for the diagnosis of NAFLD using IgM titers towards P1 was found (Fig. 3b). In addition, the measurements of IgM against P1 were divided into quartiles and the OR for NAFLD were computed across these quartiles after adjusting for age, gender, BMI, and total IgM levels (Fig. 3c, Table 3). Our results indicate that participants in quartile 1 (lowest P1-IgM levels) had an odds ratio of 5.8 (*p =* 0.01, confidence interval: 1.209–27.818), indicating an increased likelihood of NAFLD with decreasing P1-IgM levels, independent of age, gender, BMI, and total IgM levels (Fig. 3d, Table 3). Taken together, these data indicate that P1-specific IgM titers have a high predictive value for the presence of NAFLD, independent of total IgM levels.

**Table 3:** Odds ratios of P1-specific immunoglobulin M levels for non-alcoholic fatty liver disease

	Odds ratio	95 % confidence interval
Quartile 1	5.8	1.209–27.818
Quartile 2	2.431	0.613–9.647
Quartile 3	1.371	0.357–5.26
Quartile 4	1	

Adjusted for age, gender, body mass index, and total immunoglobulin M

### Anti-P1 IgM titers are not correlated with lipid levels and negatively correlate with markers for liver damage and systemic markers of inflammation

**Fig. 4:**
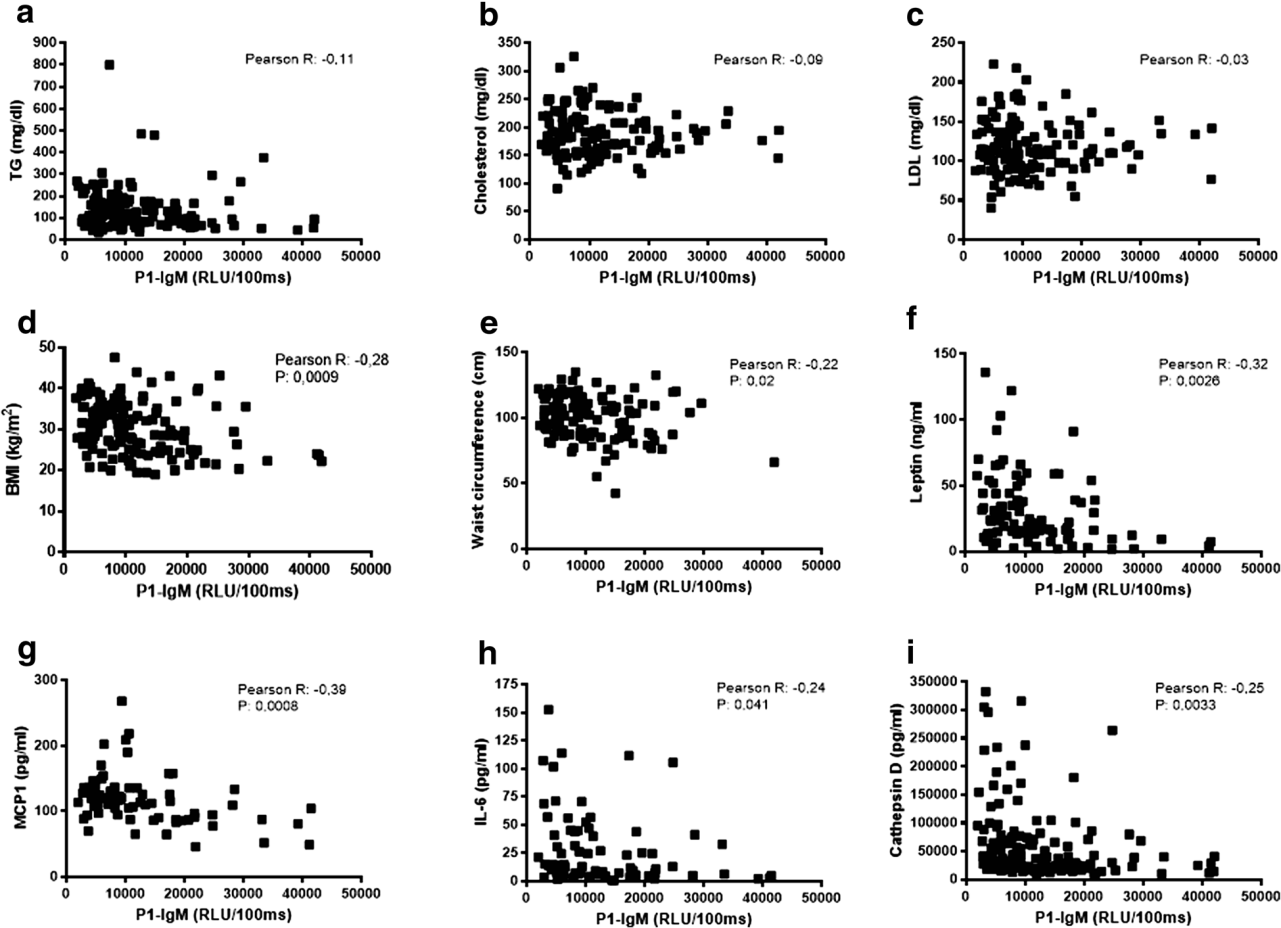
Correlation between plasma P1-immunoglobulin M (*IgM*) levels and plasma lipids and systemic markers of inflammation. Shown are the Pearson R correlation between plasma IgM titers towards P1 and **a** plasma triglycerides (*TG*), **b** cholesterol, low-density lipoprotein (*LDL*), **d** body mass index (*BMI*), **e** waist circumference, **f** leptin, and the markers for inflammation **g** monocyte chemoattractant protein 1 (*MCP1*), **h** interleukin 6 (*IL-6*), and **i** cathepsin D. *RLU* relative light unit

First, the correlation between P1-IgM and plasma lipid levels in all participants was tested. No correlation was found between IgM levels towards P1 and plasma triglycerides (Pearson R: −0.11, Fig. 4a), cholesterol (Pearson R: −0.09, Fig. 4b), or LDL (Pearson R: −0.03, Fig. 4c), well-known indicators for steatosis and risk factors for NAFLD. Next, we assessed the potential correlation with markers for obesity: BMI, waist circumference, and leptin concentration. A strong inverse correlation was observed between P1-IgM and BMI (Pearson R: −0.28, *p =* 0.0009, Fig. 4d), waist circumference (Pearson R: −0.22, *p =* 0.02, Fig. 4e), and leptin concentration (Pearson R: −0.32, *p =* 0.0026, Fig. 4f). These data are in line with a previously reported study showing elevated serum leptin levels during NAFLD [16]. Moreover, obesity, which is a known risk factor for NAFLD, has been associated with increased leptin levels [17]. Additionally, we tested the correlation between P1-IgM and circulatory markers for liver damage, inflammation, and adipokines. Interestingly, a strong inverse correlation was found between P1-IgM levels and monocyte chemoattractant protein-1 (MCP-1; Pearson R: −0.39, *p =* 0.0006, Fig. 4g), IL-6 (Pearson R: −0.24, *p =* 0.041, Fig. 4h), and cathepsin D (Pearson R −0.25, *p =* 0.0033, Fig. 4I), which we recently validated as a marker for pediatric NASH. Moreover, anti-P1-IgM titers were inversely correlated with alanine aminotransferase (ALT; Pearson R: −0.17, *p =* 0.047, Additional file 6: Figure S2A) and aspartate aminotransferase (AST; Pearson R: −0.18, *p =* 0.037, Additional file 6: Figure S2B) and a trend was observed with gamma-glutamyltransferase (γGT; Pearson R: −0.11, Additional file 6: Figure S2C). These data indicate that increased liver damage, assessed by AST and ALT levels, is associated with reduced levels of IgM antibody titers towards OSE. No correlation was found with IL-1β (Pearson R: 0.16, Additional file 6: Figure S2D) or IL-8 (Pearson R: −0.10, Additional file 6: Figure S2E). Taken together, these data suggest that immune recognition of oxidized lipids is affected during obesity and plays an important role during related diseases, such as the early stages of hepatic inflammation in NAFLD. These results also suggest an indicative role of low P1-IgM levels in regard to systemic markers of inflammation and liver damage.

### Low OSE-specific IgM levels are not associated with hepatitis C or IBD-dependent inflammation

**Fig. 5:**
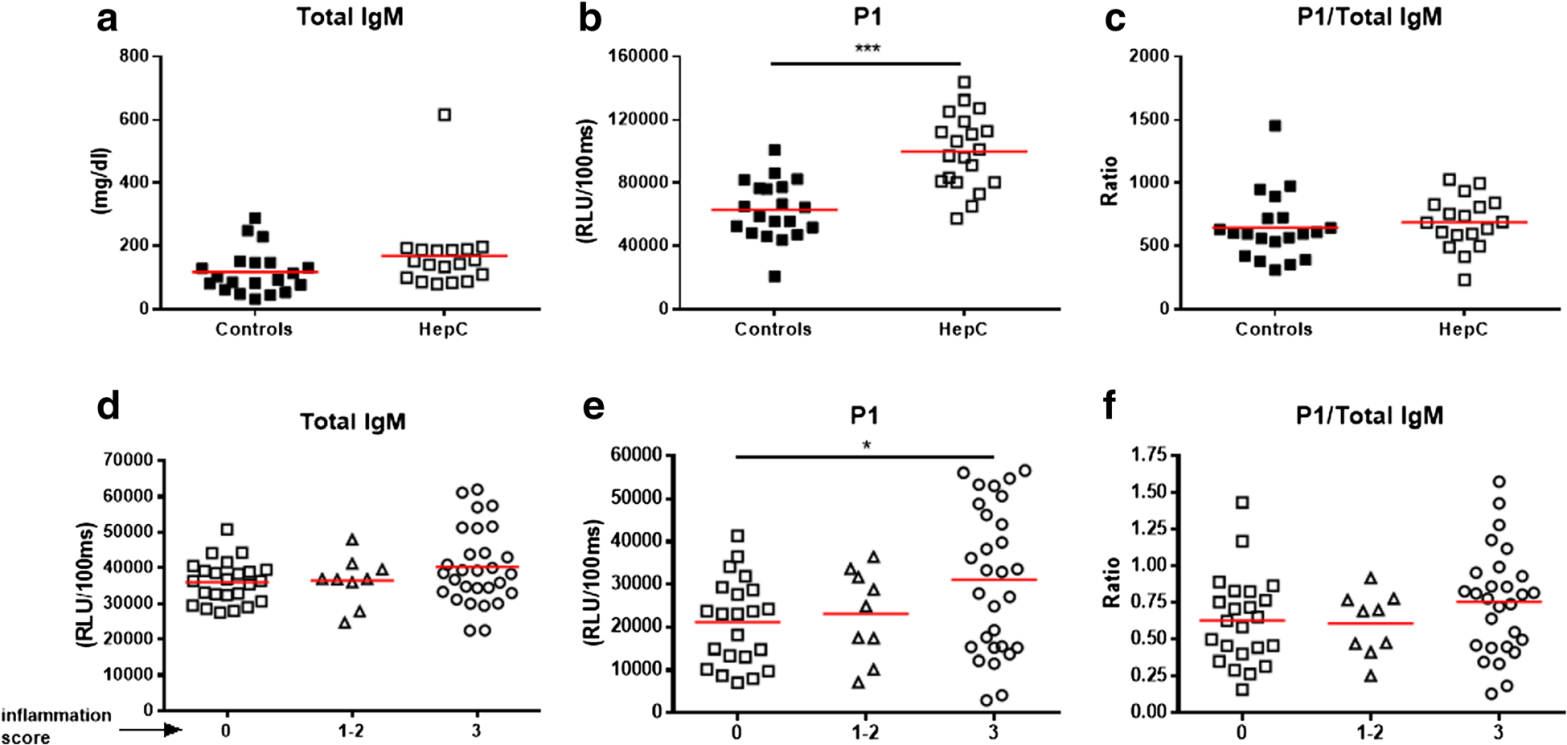
Plasma immunoglobulin M (*IgM*) titers in cohorts consisting of patients with hepatitis C or patients with inflammatory bowel disease (*IBD*). **a** Total, **b** anti-P1 IgM titers, and **c** P1/total IgM ratio in healthy controls and patients with hepatitis C. Relationship between the activity score during IBD and **d** total IgM, **e** P1-specific IgM, and **f** P1/total IgM ratio. Inactive disease was given score 0, mild to moderate disease activity was given a score of 1–2, and severe activity was given a score of 3. Data are expressed in mg/dl or relative light units (*RLU*)/100 ms and presented as means and standard deviations. *p < 0.05, ***p < 0.001

To determine the association between plasma P1-IgM levels and other chronic inflammatory diseases, a separate analysis was conducted on two cohorts consisting of patients with hepatitis C or autoimmune IBD. Total and OSE-specific IgM and IgG antibody titers were assessed in both cohorts. Total IgM titers were unchanged between patients with hepatitis C and controls (167 versus 117 mg/dL, Fig. 5a, Additional file 4: Table S7) whereas total IgG levels were higher in patients with hepatitis C than in controls (1506 versus 1091 mg/dL, Additional file 4: Table S7). Importantly, in contrast to our findings in NAFLD, IgM titers towards P1 were higher in patients with hepatitis C than in controls (99,785 versus 62,828 RLU/100 ms, *p* < 0.0001, Fig. 5b, Additional file 4: Table S7). Adjusting for total IgM antibody levels resulted in no significant differences between the two groups (686 versus 644 (ratio), Fig. 5c). OSE-specific IgG levels were higher in patients with hepatitis C than in controls (Additional file 4: Table S8). These data further suggest that low levels of IgM antibodies targeting oxidized lipids are specific for obesity-related diseases such as NAFLD.

In the IBD cohort (*n =* 62; mean male age 49.4 years and female age 54.8 years), 46 had Crohn’s disease (8 ileal, 17 colonic, and 21 ileocolonic), 15 ulcerative colitis (1 procolitis, 10 distal, and 4 pancolitis), and one patient had indeterminate colitis. IBD is characterized by fluctuations in the inflammatory state of the gut [18]. The patients were classified during endoscopy according to the activity state of inflammation at the time of blood sampling with a score ranging from 0 to 3, in which 0 means inactive (*n* = 23), 1–2 refers to mild to moderate activity (*n* = 10), and 3 indicates severely active disease (*n* = 29). In contrast to our measurements in patients with NAFLD, total IgM levels were not influenced by the inflammatory activity state in patients with IBD (36,025 versus 37,013 versus 40,280 RLU/100 ms, Fig. 5d). Next, IgM antibody titers towards the P1 mimotope were determined in these patients, reflecting the IgM titers against OSE. In contrast to the decreased levels found in the plasma of patients with NAFLD, slightly higher P1-specific IgM titers were found in the plasma of patients classified with high activity IBD (score 3) compared to patients with inactive disease (score 0) (31,052 versus 21,121 RLU/100 ms, *p =* 0.0383, Fig. 5e). We could not detect a difference in P1-specific IgM in patients scored as 0 and 1–2 (21,121 versus 23,060 RLU/100 ms, Fig. 5e), nor between patients scored as 1–2 and 3 (23,060 versus 31,052 RLU/100 ms, Fig. 5e), respectively. Additionally, no difference was observed between the groups after normalization for total IgM levels (0.628 versus 0.608 versus 0.755 (ratio), Fig. 5f). These data suggest that decreased IgM antibody titers towards MDA-type epitopes are more specifically associated with lipid-induced inflammation, as present in obesity-related diseases like atherosclerosis and in the spectrum of NAFLD.

## Discussion

The multifactorial mechanisms responsible for the worsening of NAFLD are still poorly understood. Although inflammation is believed to set the stage for further progression into fibrosis and cirrhosis, relevant insights into underlying pathways are missing. Hence, therapy options are only partially effective and accurate, non-invasive diagnostic tools to detect early signs of fatty liver disease are lacking. In the present study, we assessed the relationship between antibodies specific for model epitopes of oxidized lipids and human NAFLD. Our data indicate an inverse association between OSE-specific IgM titers and NAFLD, while no association was observed with hepatitis C or IBD.

Elevated IgM antibody levels to epitopes of OxLDL due to immunization strategies have been reported in mouse models of atherosclerosis with distinct athero-protective functions [19–22]. It has also been demonstrated that immunization with autologous MDA-LDL reduces atherosclerotic lesion formation in rabbits and mice [23, 24]. MDA epitopes were found to be highly expressed in atherosclerotic lesions [10] and antibodies recognizing immunogenic lipid peroxidation end products have also been intensively studied in patients with cardiovascular disease. IgM titers were found to be inversely associated with CVD events and low levels of OSE-specific IgM were shown to be associated with an increased risk for myocardial infarction [25, 26]. Also, during NASH, lipid peroxidation as a result of steatosis and the consequential formation of OSE such as MDA have been implicated. In addition, IgM antibodies targeting these OSE have been proposed to be protective against hepatic inflammation [8]. In line with our current data, we have previously shown that immunization of hyperlipidemic *Ldlr*^*−/−*^ mice leads to reduced hepatic inflammation upon high-cholesterol diet due to increased IgM titers towards PC, another OSE [8]. We have recently shown that deficiency of Siglec-G, a negative regulator of B-cells that results in increased B-1 cells and OSE-specific IgM antibodies, inhibits diet-induced hepatic inflammation and atherosclerosis [9]. Taken together, our current findings further support the idea that IgM targeting OxLDL and/or specific OSE play an important role during chronic fatty liver disease.

Immune reactions triggered by oxidative stress have been shown to be involved during the progression of NAFLD to fibrosis [4]. In line with data from patients with alcoholic liver disease [27], increased IgG levels against antigens derived from lipid peroxidation, such as proteins adducted with MDA, have been found in patients with established fibrosis [4]. An explanation for this association may be the potential dual effect of MDA adducts on hepatic stellate cells. It was shown that MDA is specifically recognized by hepatic stellate cells via an interaction with CD36 scavenger receptors [28], thereby potentially stimulating the production of collagen and fibronectin, and promoting an immune response towards MDA as human hepatic stellate cells can promote lymphocyte proliferation [29]. Another study demonstrated that serum IgA levels are elevated in patients with late-stage NASH in comparison with those with early-stage NASH [30]. More recently, a study by McPherson et al*.* indicated that serum IgA levels are frequently elevated in patients with NAFLD and could be useful to predict advanced fibrosis [31]. These findings were confirmed in a study by Maleki et al., showing that IgA levels are useful to evaluate the severity of liver fibrosis [32]. In both studies, total IgM levels were determined in addition to IgA levels. In line with our data, no differences were observed in the levels of total IgM antibodies in patients with late-stage NASH compared to patients with early-stage NASH, or between patients with different stages of fibrosis [30, 31]. Nevertheless, because IgM antibody titers targeting OSE were not determined in these studies it is not possible to conclude any association between levels of OSE-specific IgM to NASH development in these cohorts. Additional investigations of IgM antibodies towards different OSE, and in particular the P1 mimotope, in multiple larger cohorts are necessary to confirm the inverse correlation with NAFLD. It will be particularly interesting to follow up anti-OSE-IgM levels in patients with NAFLD and NASH who undergo treatment or lifestyle changes.

Recently, Tsiantoulas et al. showed that MDA epitopes are present on circulating microparticles and are recognized by specific IgM antibodies, thereby potentially dampening their pro-inflammatory effects [33]. Microparticles are part of a group of extracellular vesicles (0.1–1 μm in diameter) produced and released by budding from the plasma membrane upon activation or apoptosis of different cell types, including monocytes [34]. Microparticles have been found to play an important role in NAFLD and related diseases (reviewed in [35]). Patients with NASH were shown to have increased numbers of circulating microparticles from invariant natural killer T cells and macrophages/monocytes (CD14^+^), dominant cell types in mediating the pathogenesis of NASH [36]. Moreover, the level of microparticles, which was shown to correlate positively with ALT and disease severity, has been proposed as a new biomarker for NASH [36]. Another study has recently demonstrated that lipids induce microparticle release from hepatocytes, which activate macrophages and lead to an inflammatory response in NASH [37]. In line with our described findings, one may hypothesize that increased levels of microparticles due to lipid overload during the onset of NAFLD and NASH result in a consumption of OSE-specific IgM. Further studies are needed to address the role and interplay between microparticles and OSE-specific antibodies in the pathogenesis of fatty liver disease.

The similarities between atherosclerosis and NAFLD, and our observations regarding the correlations with BMI and waist circumference allow us to hypothesize that low IgM titers towards oxidized lipids are a consequence of obesity and are specifically associated with related diseases. In contrast to lipid-induced inflammation during obesity, IBD is believed to result from an inappropriate inflammatory response to intestinal microbes in a genetically susceptible host [18]. Altered serum IgG levels have previously been found in patients with IBD [38, 39]: serum IgG subclass IgG1 and IgG3 levels were elevated in ulcerative colitis, and IgG2 and IgG4 levels were increased in patients with Crohn’s disease. In line with our findings, no significant differences were found in total IgM concentration, whereas anti-OSE-IgM titers were not determined [38]. Further, different serum immunoglobulins were recently reported in patients with IBD in a cross-sectional study [40]. Similar to our observation, no correlation was found between disease activity during IBD and IgM levels [40].

Importantly, increased serum levels of MDA and lipid oxidation were found to be present in individuals with chronic hepatitis C [41], which might be reflected in a different host immune response towards epitopes of lipid oxidation, thereby limiting the specificity for our findings towards NAFLD. In line with previous reported findings, total and OSE-specific IgG antibodies were increased in patients with hepatitis C compared to healthy controls [42–45], indicative of an increased immune response towards oxidative lipids in hepatitis C. Moreover, in contrast to our findings in NAFLD, IgM titers towards P1 are increased in patients with hepatitis C compared to controls. However, adjusting for total IgM antibody levels indicates no differences between the two groups. Besides our findings in patients with IBD, these data indicate that reduced levels of IgM antibodies targeting oxidation products of lipids are not specific to fatty liver disease but rather associated with obesity-related diseases such as atherosclerosis and NAFLD/NASH. Nevertheless, additional validation in other cohorts with different chronic liver diseases is needed to support the specificity of low OSE-specific IgM titers in NAFLD.

So far, the reason behind the lower OSE-specific IgM levels in comparison to healthy participants is not clear. Similar to the view in CVD [26], one possible explanation is that, during NAFLD, there is increased consumption of IgM antibodies due to increased levels of OSE and ultimately increased clearance and/or uptake in the vessel wall. Alternatively, it is possible that genetic variation leading to lower basal IgM levels are the underlying cause for patients being at higher risk for developing lipid-induced related diseases such as atherosclerosis and NAFLD/NASH. In the latter case, low OSE-specific IgM levels should be considered a risk factor for developing fatty liver disease. Further research is needed to understand why basal differences in OSE-specific IgM levels exist and whether this can be seen as a risk factor for NAFLD.

## Conclusions

In the current manuscript we point out that IgM titers to epitopes on oxidized lipids are negatively associated with NAFLD using the P1 mimotope, which serves as a highly reproducible antigen to assess antibody titers in patients. While the link between anti-P1 IgM measurements and NAFLD, which is independent of classical risk factors for NAFLD, provides critical insights into the pathophysiology of chronic fatty liver disease, plasma levels of anti-P1 IgM are not suitable as a biomarker for the progression of NAFLD. This conclusion is based on the inability of anti-P1 IgM titers to distinguish steatosis from NASH, the correlation between P1 IgM titers to BMI, and the large overlap between NAFLD and control participants for the different measurements. Of note, our findings suggest that obesity/hyperlipidemia is a causal factor for reduced IgM levels, rendering people more prone to develop fatty liver disease and related disorders such as atherosclerosis. Additional studies assessing IgM antibodies towards different OSE, and in particular the P1 mimotope, in multiple larger cohorts are necessary to confirm the inverse correlation with NAFLD and to address the potential causal link with obesity.

## Abbreviations

ALT, alanine aminotransferase; AST, aspartate aminotransferase; AP, alkaline phosphatase; AUC, area under curve; BMI, body mass index; BSA, bovine serum albumin; CuOx-LDL, Copper oxidized low-density lipoprotein; HDL, high-density lipoprotein; IBD, inflammatory bowel disease; Ig, immunoglobulin; IL-1β, interleukin 1 beta; IL-6, interleukin 6; LDL, low-density lipoprotein; MAA, malondialdehyde-acetaldehyde; MCP-1, monocyte chemoattractant protein 1; MDA, malondialdehyde; NALFD, non-alcoholic fatty liver disease; NAS, NAFLD activity score; NASH, non-alcoholic steatohepatitis; OR, odds ratio; OSE, oxidation-specific epitope; OxLDL, oxidized low-density lipoprotein; PC, phosphocholine; RLU, relative light units; ROC, receiver operating characteristic (curve); TBS, Tris-buffered saline; γGT, gamma-glutamyltransferase.

## Additional files

Additional file 1: Data NAFLD cohort. (XLSX 1353 kb) Additional file 2: Data HepC cohort. (XLSX 28 kb) Additional file 3: Data IBD cohort. (XLS 44 kb) Additional file 4: Table S1. Shapiro–Wilk test of normality for the OSE-IgM measurements. **Table S2** Plasma total IgM and OSE-specific IgM titers in NAFLD patients and control subjects without steatosis. **Table S3** Plasma OSE-specific IgG titers in NAFLD patients and control subjects without steatosis. **Table S4** Odds ratios for the predictive power for NAFLD for different variables without adjustment. **Table S5** Odds ratios for the predictive power for NAFLD for different variables with adjustment for age, gender, BMI and total IgM levels. **Table S6** Odds ratios for the predictive power for NAFLD for different variables with adjustment for age, gender, BMI and total IgM levels. **Table S7** Plasma total and OSE-specific IgM titers in hepatitis C patients and control subjects. **Table S8** Plasma total and OSE-specific IgG titers in hepatitis C patients and control subjects. (DOCX 26 kb) Additional file 5: Figure S1. Single logistic regression model and odds ratio determination for P1-specific IgM levels in NAFLD. Odds ratios for the predictive power of anti-P1 IgM titers (log2 scale) for fatty liver disease after adjustment for age, gender, waist circumference, and total IgM. (TIF 68 kb) Additional file 6: Figure S2. Correlation between plasma P1-IgM levels and systemic markers of liver damage, inflammation, and adipokines. Pearson R correlation between plasma IgM titers towards P1 and plasma ALT (**A**), AST (**B**), γ-GT (**C**), IL-1β (**D**), and IL-8 (**E**), respectively. (TIF 110 kb)
