# Supplementary material for: Low levels of IgM antibodies recognizing oxidation-specific epitopes are associated with human non-alcoholic fatty liver disease
Source: BMC Med. 2016 Jul 22;14:107. doi: 10.1186/s12916-016-0652-0 (PMC4957359; doi:10.1186/s12916-016-0652-0)
Supplement: Additional file 4: Table S1. — Shapiro–Wilk test of normality for the OSE-IgM measurements. Table S2 Plasma total IgM and OSE-specific IgM titers in NAFLD patients and control subjects without steatosis. Table S3 Plasma OSE-specific IgG titers in NAFLD patients and control subjects without steatosis. Table S4 Odds ratios for the predictive power for NAFLD for different variables without adjustment. Table S5 Odds ratios for the predictive power for NAFLD for different variables with adjustment for age, gender, BMI and total IgM levels. Table S6 Odds ratios for the predictive power for NAFLD for different variables with adjustment for age, gender, BMI and total IgM levels. Table S7 Plasma total and OSE-specific IgM titers in hepatitis C patients and control subjects. Table S8 Plasma total and OSE-specific IgG titers in hepatitis C patients and control subjects. (DOCX 26 kb) [file 12916_2016_652_MOESM4_ESM.docx]

**Table S1: Shapiro-Wilk test of normality for the OSE-IgM measurements.**

| **Shapiro-Wilk Test of normality** | | | |
| --- | --- | --- | --- |
|  |  | **Controls** | **NAFLD** |
|  |  | ***p*-value** | ***p*-value** |
| **Untransformed** | **Cu_IgM** | 0,1219 | 0,0839 |
| **(RLU/100ms)** | **MDA_IgM** | 0,0004 | 0,0826 |
|  | **P1_IgM** | 0,0000 | 0,0000 |
|  | **MAA_IgM** | 0,0450 | 0,3536 |
|  | **PC_IgM** | 0,0000 | 0,0000 |
|  |  |  |  |
| **Log10 transformed** | **Cu_IgM** | 0,0086 | 0,0009 |
| **(Log10 RLU/100ms)** | **MDA_IgM** | 0,1359 | 0,0423 |
|  | **P1_IgM** | 0,7403 | 0,7929 |
|  | **MAA_IgM** | 0,7814 | 0,4330 |
|  | **PC_IgM** | 0,2827 | 0,0091 |

**Table S2: Plasma total IgM and OSE-specific IgM titers in NAFLD patients and control subjects without steatosis.**

|  | **Controls (*n =* 68)** | **NAFLD (*n =* 71)** | ***p*-value** |
| --- | --- | --- | --- |
| **MDA-LDL (RLU/100 ms)** | 68381 (2336) | 59005 (1890) | 0.0021** |
| **MAA-LDL (RLU/100 ms)** | 76607 (2553) | 66354 (2421) | 0.0041** |
| **P1 (RLU/100 ms)** | 14413 (1090) | 9083 (691) | 0.0009*** |
| **Cu-OxLDL (RLU/100 ms)** | 44934 (2350) | 34925(2049) | 0.0016** |
| **PC-BSA (RLU/100 ms)** | 28769 (2277) | 21848 (1398) | 0.0099** |
| **Total IgM (RLU/100 ms)** | 6123 (184) | 5391 (194) | 0.0073** |

**Table S3: Plasma OSE-specific IgG titers in NAFLD patients and control subjects without steatosis.**

|  | **Controls** | **NAFLD** | ***p*-value** |
| --- | --- | --- | --- |
| **MDA-LDL (RLU/100 ms)** | 64927 (1509) | 62348 (1472) | 0.2235 |
| **MAA-LDL (RLU/100 ms)** | 112100 (2178) | 109086 (1988) | 0.308 |
| **P1 (RLU/100 ms)** | 67017 (3678) | 69921 (3789) | 0.584 |
| **Cu-OxLDL (RLU/100 ms)** | 46116 (1584) | 42034 (1810) | 0.0947 |
| **PC-BSA (RLU/100 ms)** | 78869 (2542) | 71713 (2911) | 0.0689 |

**Table S4: Odds ratios for the predictive power for NAFLD for different variables without adjustment.**

| **Variable** | **Odds Ratio** | **95% CI for OR** | ***p*-value** |
| --- | --- | --- | --- |
| **Age** | 1.206 (per 5 year increase) | 1.039-1.4 | 0.014* |
| **Female gender** | 1.253 | 0.641-2.447 | 0.51 |
| **BMI** | 1.353 (per kg/m² increase) | 1.228-1.491 | <0.0001*** |
| **Total IgM** | 0.619 (per 1 SD increase) | 0.431-0.888 | 0.009** |
| **P1-IgM** | 0.428 (per 2-fold increase) | 0.282-0.65 | <0.0001*** |
| without adjustment |  |  |  |

**Table S5: Odds ratios for the predictive power for NAFLD for different variables with adjustment for age, gender, BMI and total IgM levels.**

| **Variable** | **Odds Ratio** | **95% CI for OR** | ***p*-value** |
| --- | --- | --- | --- |
| **Age** | 1.13 (per 5 year increase) | 0.935-1.362 | 0.207 |
| **Female gender** | 0.911 | 0.358-2.315 | 0.845 |
| **BMI** | 1.343 (per kg/m² increase) | 1.211-1.49 | 0.0001*** |
| **Total IgM** | 1.362 (per 1 SD increase) | 0.763-2.429 | 0.296 |
| **P1-IgM** | 0.419 (per 2-fold increase) | 0.216-0.813 | 0.010* |

**Table S6: Odds ratios for the predictive power for NAFLD for different variables with adjustment for age, gender, BMI and total IgM levels.**

| **Variable** | **Odds Ratio** | **95% CI for OR** | ***p*-value** |
| --- | --- | --- | --- |
| **Age** | 1.088 (per 5 year increase) | 0.904-1.311 | 0.372 |
| **Female gender** | 1.421 | 0.546-3.695 | 0.472 |
| **Waist circumference** | 1.666 (per 5 cm increase) | 1.354-2.049 | 0.0001*** |
| **Total IgM** | 1.335 (per 1 SD increase) | 0.738-2.415 | 0.339 |
| **P1-IgM** | 0.432 (per 2-fold increase) | 0.216-0.813 | 0.015* |

**Table S7: Plasma total and OSE-specific IgM titers in hepatitis C patients and control subjects.**

|  | **Controls (*n =* 20)** | **HepC (*n =* 20)** | ***p*-value** |
| --- | --- | --- | --- |
| **MDA-LDL (RLU/100 ms)** | 63407 (3928) | 67069 (4510) | 0.0408* |
| **MAA-LDL (RLU/100 ms)** | 75862 (4138) | 86337 (4643) | 0.1003 |
| **P1 (RLU/100 ms)** | 62828 (4166) | 99785 (5243) | <0.0001*** |
| **Cu-OxLDL (RLU/100 ms)** | 45191 (3298) | 63913 (4306) | 0.0013** |
| **PC-BSA (RLU/100 ms)** | 48887 (3331) | 58126 (4714) | 0.1213 |
| **Total (mg/dl)** | 117 (70) | 167 (120) | 0.12 |

**Table S8: Plasma total and OSE-specific IgG titers in hepatitis C patients and control subjects.**

| **MDA-LDL (RLU/100 ms)** | 63598 (3101) | 84493 (3055) | <0.0001*** |
| --- | --- | --- | --- |
| **MAA-LDL (RLU/100 ms)** | 102689 (2887) | 118430 (3647) | 0.0016** |
| **P1 (RLU/100 ms)** | 59099 (3894) | 76568 (4042) | 0.0036** |
| **Cu-OxLDL (RLU/100 ms)** | 52368 (1807) | 69651 (4523) | 0.0011** |
| **PC-BSA (RLU/100 ms)** | 98132 (3565) | 98066 (3958) | 0.9901 |
| **Total IgG (mg/dl)** | 1091 (44) | 1506 (74) | <0.0001*** |
