# Supplementary figures and images for: Low levels of IgM antibodies recognizing oxidation-specific epitopes are associated with human non-alcoholic fatty liver disease
Source: BMC Med. 2016 Jul 22;14:107. doi: 10.1186/s12916-016-0652-0 (PMC4957359; doi:10.1186/s12916-016-0652-0)

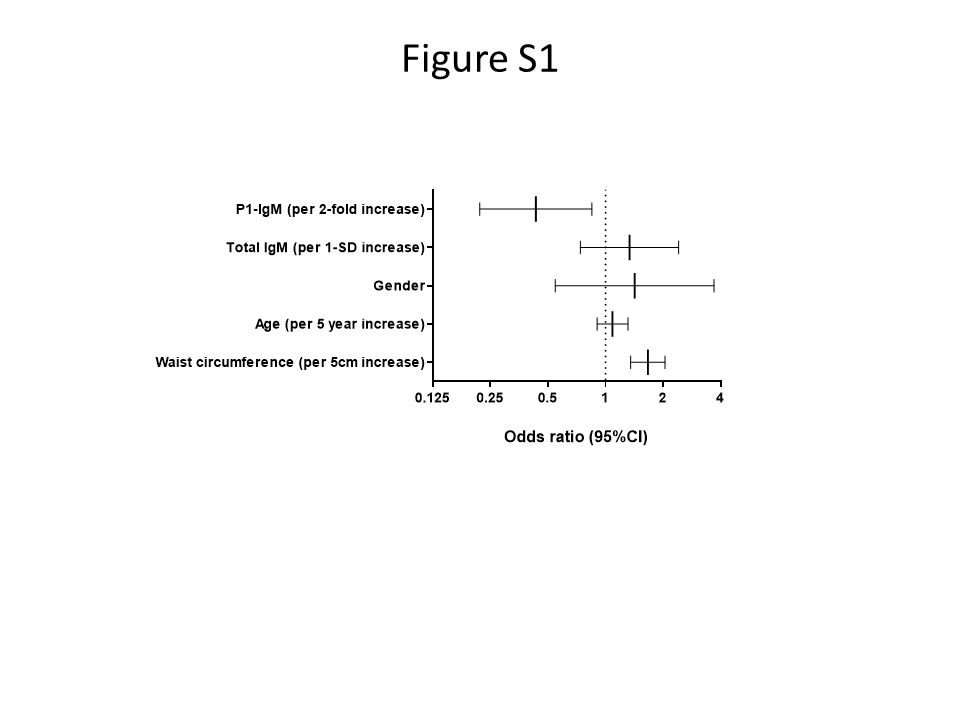

Supplement: Additional file 5: Figure S1. — Single logistic regression model and odds ratio determination for P1-specific IgM levels in NAFLD. Odds ratios for the predictive power of anti-P1 IgM titers (log2 scale) for fatty liver disease after adjustment for age, gender, waist circumference, and total IgM. (TIF 68 kb) [file 12916_2016_652_MOESM5_ESM.tif]

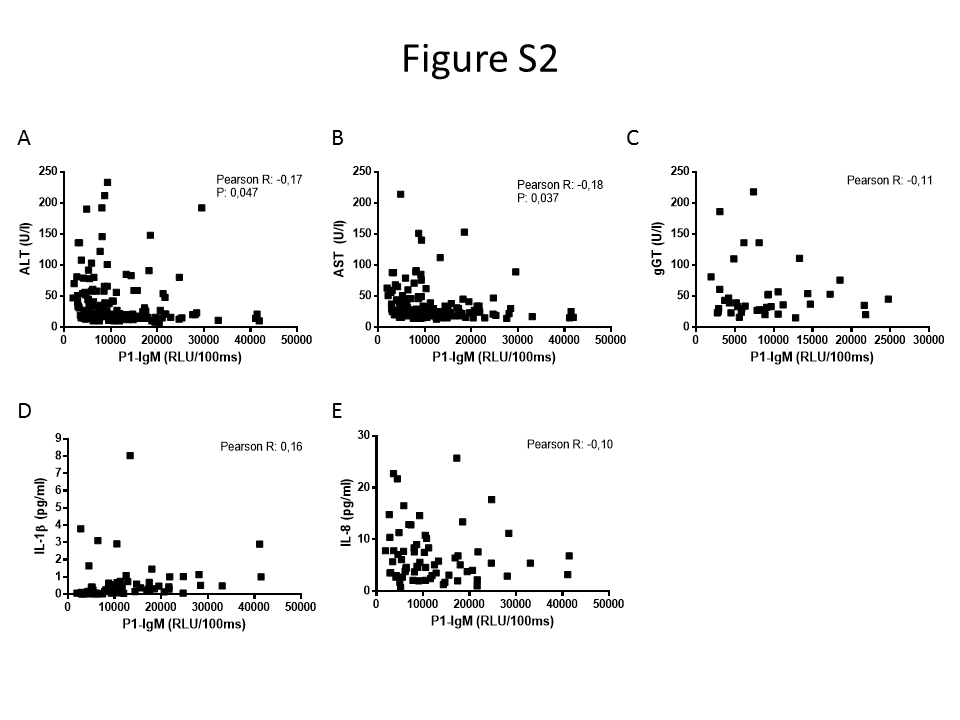

Supplement: Additional file 6: Figure S2. — Correlation between plasma P1-IgM levels and systemic markers of liver damage, inflammation, and adipokines. Pearson R correlation between plasma IgM titers towards P1 and plasma ALT (A), AST (B), γ-GT (C), IL-1β (D), and IL-8 (E), respectively. (TIF 110 kb) [file 12916_2016_652_MOESM6_ESM.tif]
